# Accuracy and role of consumer facing wearable technology for continuous monitoring during endoscopic procedures

**DOI:** 10.3389/fdgth.2024.1422929

**Published:** 2024-09-04

**Authors:** Jad P. AbiMansour, Jyotroop Kaur, Saran Velaga, Priyanka Vatsavayi, Matthew Vogt, Vinay Chandrasekhara

**Affiliations:** ^1^ Division of Gastroenterology and Hepatology, Mayo Clinic, Rochester, MN, United States; ^2^ Department of Anesthesia and Perioperative Medicine, Mayo Clinic, Rochester, MN, United States

**Keywords:** wearable electronic devices, endoscopy, gastrointestinal, remote sensing technology, monitoring, ambulatory, anesthesia

## Abstract

**Background:**

Consumer facing wearable devices capture significant amounts of biometric data. The primary aim of this study is to determine the accuracy of consumer-facing wearable technology for continuous monitoring compared to standard anesthesia monitoring during endoscopic procedures. Secondary aims were to assess patient and provider perceptions of these devices in clinical settings.

**Methods:**

Patients undergoing endoscopy with anesthesia support from June 2021 to June 2022 were provided a smartwatch (Apple Watch Series 7, Apple Inc., Cupertino, CA) and accessories including continuous ECG monitor and pulse oximeter (Qardio Inc., San Francisco, CA) for the duration of their procedure. Vital sign data from the wearable devices was compared to in-room anesthesia monitors. Concordance with anesthesia monitoring was assessed with interclass correlation coefficients (ICC). Surveys were then distributed to patients and clinicians to assess patient and provider preferences regarding the use of the wearable devices during procedures.

**Results:**

292 unique procedures were enrolled with a median anesthesia duration of 34 min (IQR 25–47). High fidelity readings were successfully recorded with wearable devices for heart rate in 279 (95.5%) cases, oxygen in 203 (69.5%), and respiratory rate in 154 (52.7%). ICCs for watch and accessories were 0.54 (95% CI 0.46–0.62) for tachycardia, 0.03 (95% CI 0–0.14) for bradycardia, and 0.33 (0.22–0.43) for oxygen desaturation. Patients generally felt the devices were more accurate (56.3% vs. 20.0% agree, *p* < 0.001) and more permissible (53.9% vs. 33.3% agree, *p* < 0.001) to wear during a procedure than providers.

**Conclusion:**

Smartwatches perform poorly for continuous data collection compared to gold standard anesthesia monitoring. Refinement in software development is required if these devices are to be used for continuous, intensive vital sign monitoring.

## Introduction

The term wearable technology encompasses a wide spectrum of devices worn on or near a person (1). Market research suggests that one in five Americans are users of wearable technology with the market currently valued at over 30 billion dollars annually (2, 3). Current technology allows consumer-facing wearables to record physiologic information, ranging from heart rate to pulse oximetry, as well as audio and video. These devices are often marketed to improve health, and there has been significant interest in their use to diagnose, monitor and treat disease (4). This includes diverse conditions ranging from atrial fibrillation (5) to menstrual cycle length (6).

The interest in wearable technology for remote health monitoring was accelerated by the COVID-19 pandemic which strained ambulatory and inpatient resources, further highlighting an opportunity to provide monitoring and care in non-traditional healthcare settings, including patients' homes (7). Beyond clinical monitoring, wearable technology is poised to impact biomedical research by facilitating remote clinical trials. The decentralization of trials would not only make participation less burdensome to patients but also allow a more holistic understanding of disease process and impact on patients' daily lives (8). Patients who own wearable devices have also been shown to be quite interested in sharing and using of their data for medical research (9).

However, studies of consumer-facing products have raised questions regarding accuracy and reliability for intensive monitoring of vital signs. There have also been reports of unintended interference from consumer wearables, resulting in disabled clinical devices (10, 11). A temporary guidance document issued by the Food and Drug Administration (FDA) further highlighted the need to rigorously evaluate the reliability and accuracy of remotely collected data obtained during clinical trials (12). The aim of the current study is to evaluate the accuracy and reliability of readily available consumer-facing wearable technology for intensive continuous monitoring compared to gold-standard anesthesia monitoring in a controlled clinical environment. Patient and provider preferences around the use of these devices in clinical environments was also assessed.

## Methods

A prospective, single-arm study of adult patients undergoing endoscopy with anesthesia support from June 2021 to June 2022 was performed. The protocol was approved by the Mayo Clinic Institutional Review Board (#21-007738). Patients were identified prior to the procedure. Baseline demographic information including age, gender, body mass index (BMI), and Fitzpatrick skin tone was recorded (13). Prior to undergoing the clinically-indicated endoscopic procedure, participants were provided with a smartwatch (Apple Watch Series 7, Apple Inc., Cupertino, CA). This device manufacturer was selected due to having the largest international market share and the most likely to be encountered in a clinical setting (14). Smartwatch monitoring capabilities were augmented with the use of commercially-available accessories which consisted of a continuous ECG monitor and pulse oximeter (Qardio Inc., San Francisco, CA). All device data was stored on a paired smartphone device which traveled with the patient and was retrieved after the procedure. Data was also simultaneously obtained from the in-room anesthesia monitor (Aisys CS2, GE HealthCare, Chicago, IL) during the procedural anesthesia encounter.

Vital sign data was evaluated in 3 domains consisting of heart rate, oxygenation, and respiration using the manufacturer-recommended settings. The Apple Watch generated continuous heart rate readings and background arrhythmia monitoring; however other sampling frequencies for measurements like oxygenation or respiratory rate were not able to be determined or adjusted due to the closed nature of the operating system. The accessories allowed continuous measurement of heart rate, respiratory rate and oxygenation with manufacturer reported frequency ranging from 0.05 to 40 Hz. Data were extracted using the iPhone Health app and QardioMD portal and manually reviewed. Vital signs recorded by clinical devices were monitored real time in the procedure room. Both absolute values (i.e., minimum and maximum heart rate, oxygen saturation, and respiratory rate) and events (i.e., tachycardia, bradycardia, desaturation, tachypnea, bradypnea) were recorded. The definition of these events is outlined in Table 1. Thresholds were chosen to reflect clinically significant derangements based on previously validated thresholds defined in resuscitation literature as “nonroutine events” (15). Arrythmia detection was also captured, defined as any irregular rhythm interrupting normal sinus rhythm. Concordance of the absolute maximum and minimum values as well as number of nonroutine events using anesthesia monitoring as the gold standard was assessed with interclass correlation coefficients (ICC) using a two-way mixed effect, single rater model (16). Poor reliability was defined by ICC 0–0.5, moderate reliability 0.5–0.75 and good reliability 0.75–0.90 (17).

**Table 1:** Definition of vital sign events.

Event	Definition
Tachycardia	Heart rate >130
Bradycardia	Heart rate <50
Desaturation	Oxygen saturation <90%
Tachypnea	Respiratory rate >30
Bradypnea	Respiratory rate <6

Survey questionnaires were administered including 8 questions for providers and 15 questions for patients. Questionnaires were designed and distributed via an electronic research database manager (REDCap, Nashville, TN) hosted at our institution after the procedures were completed (Supplementary File). The survey was distributed to anesthesia and endoscopy providers who performed procedures on patients enrolled in the study, including gastroenterologists, anesthesiologists, and certified registered nurse anesthetists. Agreement with various prompts were assessed using a 5-point Likert scale, ranging from strongly disagree to strongly agree. Analysis was performed by categorizing responses as agreement (strongly agree or agree), neutrality (neutral), or disagreement (disagree or strongly disagree) and comparing patient and provider responses using Chi-squared testing. Significance was defined by a *p*-value <0.05.

## Results

A total of 290 patients undergoing 292 unique procedures were enrolled in the study (Table 2). The majority of cases were esophagogastroduodenoscopy (EGD) alone (151, 51.7%) with 94 (32.2%) colonoscopies, and 20 (6.8%) combined EGD and colonoscopy. Most cases were performed as monitored anesthesia care (MAC) with propofol (242, 82.9%) with a median anesthesia time of 34 min (IQR 25.0–47.0). The smartwatch alone was able to record high fidelity readings for heart rate in 279 (95.6%) of cases and oxygen saturation in 14 (4.8%). No evident measurement of respiration rate was obtained from the watch alone in any case. The accessories alone were able to provide heart rate readings in 143 (49.0%) cases, oxygen saturation in 203 (69.5%), and respiratory rate in 154 (52.7%).

**Table 2:** Patient and characteristics of each unique procedure.

	*N* = 292
Age, median (IQR)	60.0 (45.8–71.0)
Females, *n* (%)	173 (59.2)
Fitzpatrick skin tone	
1–2, *n* (%)	255 (87.3)
3–4, *n* (%)	31 (10.6)
5–6, *n* (%)	6 (2.1)
BMI, median (IQR)	28.3 (24.1–33.2)
Procedure	
EGD, *n* (%)	151 (51.7)
Colonoscopy, *n* (%)	94 (32.2)
EGD/Colonoscopy, *n* (%)	20 (6.8)
Other (EUS, ERCP, enteroscopy), *n* (%)	27 (9.2)
Procedure duration, min, median (IQR)	15.0 (8.0–26.0)
Anesthesia duration, min, median (IQR)	34.0 (25.0–47.0)
Anesthesia	
Moderate sedation, *n* (%)	2 (0.7)
MAC, *n* (%)	242 (82.9)
General, *n* (%)	46 (15.8)
No sedation, *n* (%)	2 (0.7)

EGD, esophagogastroduodenoscopy; EUS, endoscopic ultrasound; ERCP, endoscopic retrograde cholangiopancreatography; MAC, monitored anesthesia care.

**Figure 1:**
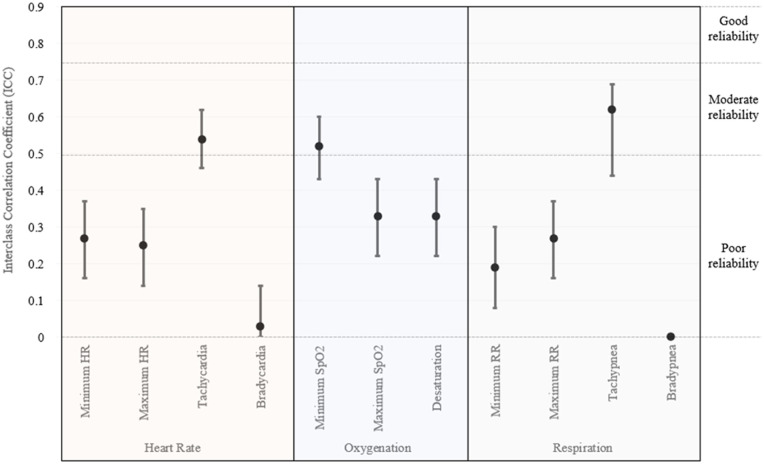
Interclass correlation coefficients for vital sign monitoring utilizing smartwatch accessories.

Interclass correlation coefficients for the smartwatch alone were 0.73 (95% CI 0.67–0.78) for minimum heart rate, 0.48 (95% CI 0.39–0.57) for maximum heart rate, 0.37 (95% CI 0.27–0.47) for bradycardia, and 0 for tachycardia (95% CI 0–0.12). ICC were not able to be calculated for the other vital sign domains due to a lack of adequate readings. ICC for vitals from included accessories are shown in Figure 1. The highest ICC was noted for the detection of tachycardia (0.54, 95% CI 0.46–0.62), tachypnea (0.62, 95% CI 0.44–0.69), and minimum oxygen saturation (0.52, 95% CI 0.43–0.60); however, the remainder of the vital sign measurements were below 0.5. No arrythmias were detected by gold-standard anesthesia monitoring or any device in the enrolled cohort.

An electronic survey was distributed to 290 patients and 22 providers. A total of 126 patients (43.4%) and 15 providers (68.2%) completed the survey. Of the 126 patients who responded, 48 (38.1%) indicated that they personally utilize a smart watch and 85 (67.5%) agreeing that smartwatches were useful for general wellness. In specific regards to wearing smartwatches during an endoscopic procedure, most patients (114, 90.5%) described that the devices as comfortable to wear and 106 (84.1%) requesting to wear them during future procedures if given an opportunity.

When asked if the majority of patients own smartwatches, 47.9% patients agreed vs. 6.7% of providers (*p* < 0.001). Patients were more likely to feel that they should have an option to wear smart devices during procedures (56.3% vs. 33.3% agreement; *p* < 0.001). When compared to their providers, more patients also felt that smart watches could accurately measure vital signs such as heart rate and pulse oximetry (59.5% vs. 20.0% agreement; *p* < 0.001) and felt the devices were important for health monitoring (80.2% patients vs. 20.0% of providers; *p* < 0.001).

## Discussion

Consumer-facing wearable devices are distinct from the typical devices utilized in carefully regulated healthcare settings. However, with increasing sophistication and imbedded technology, these devices are now marketed for the monitoring and treatment of specific diseases with FDA approval. Specifically, the irregular rhythm notification on the Apple Watch has FDA clearance for (1) the ECG application in patients 22 years and older (18) and (2) irregular rhythm notification in patients older than 22 years old without a history of atrial fibrillation (19). The accessories utilized in this study are approved for ECG recording periods of up to 24-h in a single session in adult patients who may be asymptomatic or who meet clinical indications to perform an ECG-Holter monitor exam (20). The oxygenation and respiratory rate features were not FDA cleared on any of the devices.

These devices and onboard software are generally intended to supplement the remote monitoring of ambulatory patients. There are approved indications for intensive clinic monitoring, and this study is not designed to assess or refute current market claims. Rather, these results provide exploratory insight into the performance of these increasingly prevalent and affordable devices in a novel setting which may be of interest to patients, clinicians, and institutions. This is particularly true in the setting of endoscopy where patients are routinely allowed to wear their smart devices during their procedure and may become curious about physiologic aberrancies noted on their personal device.

There has been extensive work on vital sign monitoring using consumer-facing devices. Data exists to support their technical capabilities and accuracy, however, there remains significant variation in the literature. Studies comparing the Apple Watch Series 6 to medical grade pulse oximeters reported ICC ranging from 0.76 to 0.89 with a meta-analysis of 5 studies suggesting a limit of agreement ranging from 2.7% to 5.9%, with some variation reported up to 15% (21). Another study evaluated the accuracy of heart rate measurement and reported a correlation coefficient of 0.7 when compared to standard-of-care telementry (22). A study of several commercially available wrist-based devices showed accurate heart rate readings from 6 of 7 manufacturers (defined as a mean error <5%) with the Apple Watch showing the lowest rate of overall error (23). Another study evaluated accuracy of HR and SpO2 for four devices reported a Pearson correlation coefficient of 0.77 when compared to clinical devices (24). There have not been reports of consumer-facing devices accurately measuring respiratory rate, although dedicated devices worn on the chest have shown promising results (25).

However, it should be noted that these studies have largely focused on passive monitoring of ambulatory patients to detect an abnormality (5) or concatenate data for later review (26). These studies generally employ single, active measurements (e.g., obtaining a single oxygen saturation measurement and comparing at a single time point) or long-term surveillance (e.g., monitoring for over 3 months to detect atrial fibrillation). This study was conducted in a unique environment and highlights how these devices may perform in a controlled, clinical context where real-time vital sign monitoring is required.

Results from this exploratory study suggest poor performance across all domains evaluated including heart rate, oxygenation, and respiration with correlation coefficients well below what would be considered adequate. While this is likely driven by several factors, a large component is rooted in the devices' closed ecosystems and limited ability to modify when, how, or why recordings are obtained and stored. This was somewhat anticipated during study design which prompted the inclusion of commercially available accessories, allowing for the recording of additional vital signs which were not captured on the watch alone, namely oxygenation and respiration. However, even with the inclusion of these dedicated accessories, vital sign readings remained discordant from gold standard clinical devices. Wearable devices, including the ones studied here, consist of both hardware as well as software which contribute to functionality and intended use. That is, a device may contain a medical grade pulse oximeter, but key software is needed to interpret, display, and transmit the results. Many are familiar with the FDA's role in physical hardware/device regulation but software can also be classified as a medical device.

The hardware on board consumer-facing devices is likely sufficient to provide accurate readings as prior data suggests, but the software likely requires significant adjustments if it is to be used in real-time clinical environments. The FDA has issued guidance on the clinical validation of software as a medical device and emphasizes three tenets: (1) valid clinical association between software output and a clinical condition, (2) analytic variation—the accurate processing of input to accurate, reliable, and precise outputs, and (3) validation that the output data achieves intended purpose (27). Further refinement of these devices and investigation with this framework is required before bring them into a clinical setting.

It would be reasonable to note that smartwatches and other consumer-facing wearables were never intended to perform real-time vital sign monitoring, which may explain the discrepancy in accuracy seen in this study. However, patient perception continues to show a high degree of belief in the accuracy of the devices and desire to wear them during procedures, which differs significantly from their providers. The medicolegal implications of device recording in environments like the endoscopy suite, where unlike operating rooms patients often maintain possession of personal devices, remain undefined. As vital sign readings obtained do not seem to readily correlate with clinical-grade equipment, inaccurate data may cause unnecessary stress to patients as well as unnecessary risk to institutions. The ability for wearable devices to record audio and video also raises administrative and medicolegal questions regarding their permissibility in clinical contexts. It has been suggested that units have policies in place to address wearable technology and ensure they are consistently enforced (28). Data from this study can help inform such policies, as well as discussions with patients in the preprocedural area. A multidisciplinary approach, including patients and their advocates, when designing wearable-related policies would be beneficial given the positive way the technology is viewed from a patient standpoint.

From remote monitoring of chronic conditions to decentralized clinical trials, understanding the limitations of continuous vital sign monitoring is essential as expanding use cases for these devices are explored. Current design favors on demand measurements rather than high frequency or continuous monitoring. That is, a smartwatch may be adequate to evaluate a patient's heart rate trend over the course of several days but may be ill-equipped to assess a response shortly after consuming a study drug or performing an intervention. Other limitations include physical reliability to keep devices fixed during movement and/or repositioning, indicators of reliability or how well the device is performing over a certain time interval, and minimizing the need for multiple separate accessories Accordingly, many remote monitoring programs have shifted away from the use of consumer-facing devices in favor of medical-grade, Bluetooth-enabled platforms which are now available from several biotechnology companies (29). There have also been significant advances in non-contact sensing technology which may address limitations created by physical sensors that must be worn on a patient's body (30, 31).

Limitations of this study include the use of a single smartwatch manufacturer with numerous other devices on the market. As noted, the device used in this study was chosen due having the largest market share and augmented with accessories. However, it should be acknowledged that other devices may perform better for continuous monitoring. There were an insufficient number of events to comment on arrythmia detection which is one of the FDA-approved indications for the device. Almost all patients in the study underwent sedation with propofol, and external validity for continuous monitoring without sedation or conscious sedation is limited. Similarly, this study was performed in an endoscopy unit, but the results are likely applicable to multiple other healthcare environments contexts.

Smartwatches, despite dedicated health monitoring accessories, were shown to perform poorly for passive continuous data collection when compared to gold-standard anesthesia monitoring in patients undergoing endoscopy with anesthesia-assisted sedation. This is a scenario clinicians may experience given the increasing prevalence of smart devices, and patients should be cautioned when interpreting aberrant readings that may have been generated during a procedure. Continued iteration and development of these devices, with particular attention to software, may enhance monitoring capabilities in the future; however, the current state is likely insufficient to employ clinically. Particular attention should be given to differences in patient and provider perceptions as these devices become more commonplace and institutions seek to implement policies regarding their use.

## Data Availability

The raw data supporting the conclusions of this article will be made available by the authors, without undue reservation.
