# Supplementary material for: Accuracy and role of consumer facing wearable technology for continuous monitoring during endoscopic procedures
Source: Front Digit Health. 2024 Sep 4;6:1422929. doi: 10.3389/fdgth.2024.1422929 (PMC11443421; doi:10.3389/fdgth.2024.1422929)
Supplement: Supplementary file 1 [file Datasheet1.pdf]

Procedure Smart Watch Survey

Thank you for recently participating in our study regarding smart watches and endoscopic procedures.

We would like to ask you a few questions regarding smart watches like the Apple Watch. Please complete the survey below.

Procedure Experience

		0	1	2	3	4	5	6	7	8	9	10
1)	What was your level of awareness during your procedure? (if 0 is not aware at all and 10 is fully aware)	○	○	○	○	○	○	○	○	○	○	○
2)	What was your level of discomfort or pain during your procedure? (if 0 is none at all, and 10 is the worst possible)	○	○	○	○	○	○	○	○	○	○	○

		Strongly disagree	Disagree	Neither agree or disagree	Agree	Strongly Agree
3)	The smart watch was easy to wear	○	○	○	○	○
4)	The smart watch was comfortable to wear	○	○	○	○	○
5)	The smart watch did not interfere with my procedure	○	○	○	○	○
6)	If given the opportunity, I would wear a smart watch for monitoring during future procedures	○	○	○	○	○

7) How satisfied were you with wearing the watch during your procedure

○ Very dissatisfied ○ Dissatisfied ○ Neither satisfied or dissatisfied ○ Satisfied ○ Very satisfied

		Strongly disagree	Disagree	Neither agree or disagree	Agree	Strongly agree
8)	Most people have wearable smart devices (like a watch)	○	○	○	○	○
9)	Smart watches are useful for general wellness	○	○	○	○	○
10)	Smart watches can accurately measure things like heart rate and oxygen levels	○	○	○	○	○
11)	Patients should have the option to wear smart devices like a watch during their procedures	○	○	○	○	○

12) Do you personally use a smart watch? (eg. Apple Watch or Fitbit)? ○ Yes ○ No

If yes, how important are the following features?

		1 - Not important at all	2	3	4	5 - Very important
13)	Activity measurement (steps, standing/sitting, sleep)	○	○	○	○	○
14)	Health monitoring (heart rate, oxygen, heart rhythm)	○	○	○	○	○
15)	Communication (calls, texts)	○	○	○	○	○

Wearable Tech Provider Survey

Thank you for playing a role in the Wearable Technology project. Your response to this very brief, 8 question survey would be greatly appreciated.

1) Department

◯ Gastroenterology
◯ Anesthesia

Provider Perspectives on Consumer-Facing Wearable Technology

	Strongly disagree	Disagree	Neither agree or disagree	Agree	Strongly agree
2) Most of my patients have a smart watch	◯	◯	◯	◯	◯
3) Patients should have the option to wear smart devices like a watch during their procedures	◯	◯	◯	◯	◯
4) I would feel comfortable with a patient wearing a smart watch during a procedure	◯	◯	◯	◯	◯
5) I am aware of Mayo Clinic policies regarding smart devices in procedural areas	◯	◯	◯	◯	◯
6) Mayo Clinic should have a policy regarding the use of wearable smart devices in procedural areas	◯	◯	◯	◯	◯
7) Smart watches can accurately measure vital signs like heart rate and oxygen levels	◯	◯	◯	◯	◯
8) Smart watches can be used to monitor a patient during a procedure	◯	◯	◯	◯	◯
9) Smart watch data would be permissible evidence in a court room (for example, a malpractice case)	◯	◯	◯	◯	◯
